# Prediction of CRISPR-Cas9 off-target activities with mismatches and indels based on hybrid neural network

**DOI:** 10.1016/j.csbj.2023.10.018

**Published:** 2023-10-16

**Authors:** Yanpeng Yang, Jian Li, Quan Zou, Yaoping Ruan, Hailin Feng

**Affiliations:** a School of Mathematics and Computer science, Zhejiang A&F University, Hangzhou 311300, China; b Yangtze Delta Region Institute (Quzhou), University of Electronic Science and Technology of China, Quzhou 324000, China; c Institute of Fundamental and Frontier Sciences, University of Electronic Science and Technology of China, Chengdu 610054, China

**Keywords:** CRISPR-Cas9, Off-target prediction, Hybrid neural network, Encoding scheme

## Abstract

The CRISPR/Cas9 system has significantly advanced the field of gene editing, yet its clinical application is constrained by the considerable challenge of off-target effects. Although numerous deep learning models for off-target prediction have been proposed, most struggle to effectively extract the nuanced features of guide RNA (gRNA) and DNA sequence pairs and to mitigate information loss during data transmission within the model. To address these limitations, we introduce a novel Hybrid Neural Network (HNN) model that employs a parallelized network structure to fully extract pertinent features from different positions and types of bases in the sequence to minimize information loss. Notably, this study marks the first application of word embedding techniques to extract information from sequence pairs that contain insertions and deletions (Indels). Comprehensive evaluation across diverse datasets indicates that our proposed model outperforms existing state-of-the-art prediction methods in off-target prediction. The datasets and source codes supporting this study can be found at https://github.com/Yang-k955/CRISPR-HW.

## Graphical abstract

**Figure:**
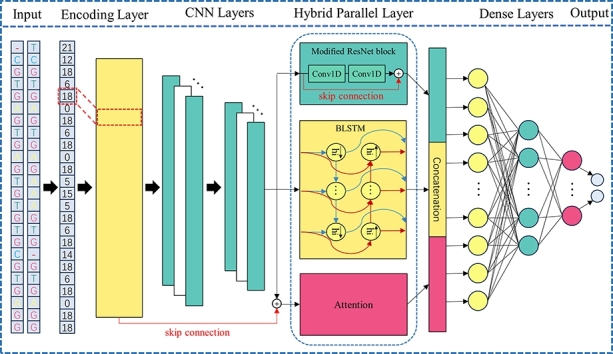

## 1 Introduction

**Fig. 1:**
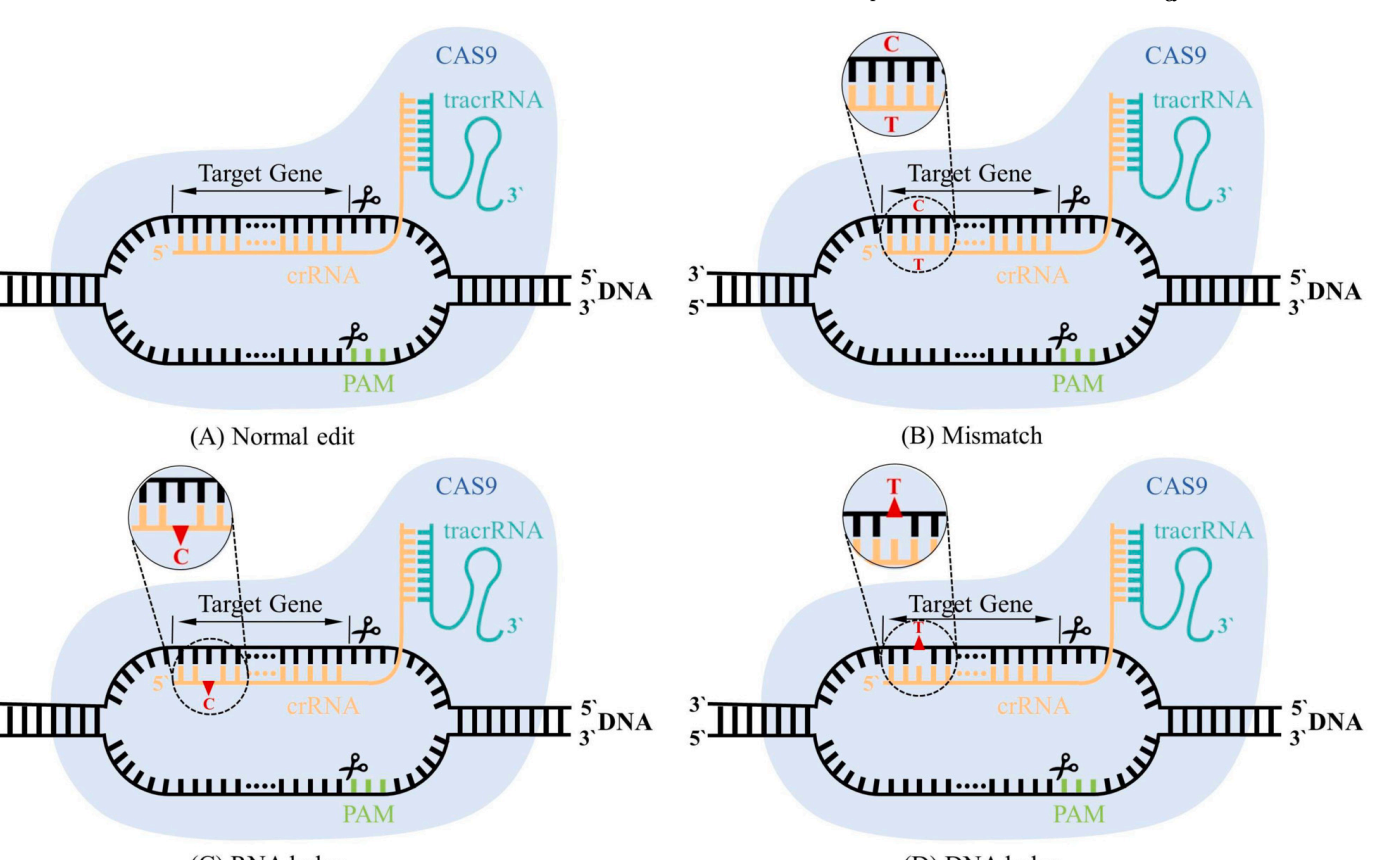
The CRISPR-Cas9 system and the three types of off-target. (A) is the normal gene editing process. The gRNA formed by the binding of crRNA and tracrRNA searches for a target site in the target DNA, and after a successful match, the Cas9 protein acts as its endonuclease and cleaves the target DNA sequence after PAM. (B) is the base mismatch in the three off-target cases. Similarities between target sequences and other genomic regions may cause Cas9 proteins to bind and cleave at the wrong location, thus triggering off-target effects. (C) is the bulge case of sequence RNA. (D) is the bulge case of sequence DNA.

CRISPR-Cas9 (Clustered Regularly Interspaced Short Palindromic Repeats and CRISPR-associated Protein 9) is a revolutionary genome-editing technology that has gained widespread use in genetic engineering and gene therapy due to its simplicity, efficiency, and relative precision [1]. This system is shown in Fig. 1, composed of unique DNA sequences found in bacteria and archaea, has three primary components: the Cas9 protein, a chimeric single-guide RNA (gRNA), and a protospacer adjacent motif (PAM). The length of 20 nucleotides of gRNA is a synthetic RNA which combines CRISPR RNA (crRNA) and trans-activated crRNA (tracrRNA). crRNA binds to the DNA of the target site, and the latter tracrRNA can interact with Cas9 nuclease to form a ribonucleoprotein complex used to locate the target site and for double-strand break. The PAM, a three-nucleotide motif, is located in close proximity to the target site and is essential for Cas9 protein cleavage [2].

CRISPR-Cas9 gene editing technology has revolutionized the field of genetics by enabling precise and targeted modifications of DNA sequences. However, one of the major challenges associated with CRISPR-Cas9 gene editing is the potential for off-target mutations [3], [4]. Off-target mutations occur when the Cas9 protein cleaves DNA sequences which are similar to, but not identical to, the intended target DNA sequence. These include mismatches, RNA bulges and DNA bulges of gRNA-DNA target [5], [6]. Off-target effects pose significant safety and efficacy concerns for clinical applications of the CRISPR-Cas9 system. Therefore, the development of an efficient and robust prediction model is essential to improve traditional off-target prediction methods, as this can reduce off-target effects and improve accuracy.

Historically, the detection of off-target effects in CRISPR gene editing predominantly relied on manual techniques that were laborious and time-consuming. These techniques encompassed both in vitro and in vivo assays, in the realm of in vitro assays, techniques such as TC-seq (Targeted Cleavage by sequencing) [7], Digenome-Seq [8], and CIRCLE-Seq [9] were employed; In the case of in vivo assays were also utilized to assess off-target effects, such as ChIP-Seq (Chromatin Immunoprecipitation sequencing) [10], GUIDE-Seq (Genome-wide, Unbiased Identification of DSBs Enabled by sequencing) [11], HTGTS (High-Throughput Genome-wide Translocation Sequencing) and IDLVs (Integration-Deficient Lentiviral Vectors) [12]. With the advent of computational methods in bioinformatics, researchers have shifted towards lower cost and more efficient methods to predict off-target effects. As such, computational methods have emerged as the preferred choice for off-target prediction due to their superior efficacy, accuracy, and speed. This discussion will primarily focus on these cutting-edge in silico methods, which can be broadly classified into two categories: those utilizing conventional machine learning methodologies and those employing advanced deep learning techniques.

At the target site, accurate prediction of gRNA off-target activity necessitates meticulous data preprocessing prior to model training, and the crucial step in this preprocessing is the conversion of gRNA-Target sequence pairs into Fixed dimensional feature vectors which can be comprehended and interpreted by the predicting model. The sequence coding techniques currently applied to the above-mentioned conversion fall into two main categories: one-hot coding and word embedding. In the one-hot coding scheme, to encode each gRNA and DNA sequence, a one-hot matrix is utilized, which consists of four rows representing the four bases (adenine, thymine, guanine, and cytosine) and L columns corresponding to the length of the sequences. For example, Chuai et al. [13] proposed DeepCRISPR, the first to apply deep learning models for off-target detection in CRISPR applications, where the gRNA and DNA sequences were respectively encoded as a independent matrix of (4+n)*23 (Here, n represents the number of epistatic heritage features). However, the fusion of the two matrices was not conducted before training the model, preventing the learning of inter-base pair roles. For this drawback, Lin et al. [14] proposed a fusion technique utilizing the OR operation to combine the two one-hot matrices, and the resulting fused matrix can reflect the base pairs relationship in each gRNA-Target sequence pair. Yet, this simple fusion led to the loss of information, as it fails to distinguish the orientation of the base pairs, such as ‘AT’ and ‘TA’, the base pairs switched orientation while their codes remained the same. To capture the orientation information between base pairs, Charlier et al. [15] spliced two 4*23 matrices into an 8*23 matrix. Although the aforementioned one-hot coding methods showed promising results, they did not account for the off-target activity resulting from insertions or deletions (Indels) between base pairs. Lin et al. [16] introduced a novel coding scheme that utilizes the one-hot method to convert DNA or RNA bulges into a 7*23 binary matrix comprising seven-bit channels. This coding scheme employs five bits to represent nucleotides at the on-target site and the off-target site, while the remaining two bits are used to identify the type of insertion, deletion or mismatch. Nonetheless, these encoded data by one-hot tend to lose some information owing to the variable size of the convolution kernel employed by the model [17]. In the word embedding coding scheme, researchers usually convert gRNA-Target sequence pairs into high-dimensional vectors by different methods of word embedding. For example, Liu et al. [18] employed the Glove [19] model to convert sequences into vectors; Zhang et al. [20] utilized Word2Vec technique to encode base pairs and mapped to a high-dimensional [21]. While these methods leverage word embedding, they do not encompass all three cases of Off-Target Activities.

In terms of models for off-target prediction, several network models have been developed, with the most popular being Feedforward Neural Networks (FNNs) [22], Convolutional Neural Networks (CNNs) [23], Recurrent Neural Networks (RNNs) and Transformer [24]. FNN represent the most fundamental form of neural networks, composed of an input layer, several hidden layers, and a final output layer. In contrast, CNNs and RNNs are more complex and powerful models. CNNs use convolutional kernels of different sizes to process local features of the sequence, followed by fully connected layers for further processing. RNNs, such as LSTM [25] and GRU [26], can capture the temporal dependencies in sequence data and learn contextual features. The Transformer network model has emerged as a powerful architecture for handling sequential data, the attention mechanism in the Transformer network enables the model to capture long-range dependencies and contextual information effectively. Although applying various network models simultaneously for off-target prediction, good performance can be achieved, the current off-target dataset has positive and negative sample imbalance, the deeper layers of the model network and the more features of the original data are lost. Existing studies have indicated that residual block [27] and parallelizing the network model can effectively alleviate this problem [28], but the application of residual block and parallelized HNN models remains unexplored in off-target prediction.

Although significant progress has been made in predicting off-target effects through various coding schemes and models, several challenges persist. In terms of encoding strategies, the prevalent one-hot encoding scheme suffers from information loss regarding the relationships between base pairs. On the other hand, the word embedding encoding scheme fails to account for the three distinct off-target cases. Regarding the models themselves, the presence of imbalanced data and the increasing complexity of deeper models hinder effective learning of features from negative samples. Consequently, there is a clear need for further refinement and enhancement of the current coding methods and off-target prediction models.

To overcome these deficiencies, we propose an encoding scheme that applies a word embedding technique to capture the insertions, deletions, and mismatched off-target activities between base pairs of sequence, and then maps them to a dense real-valued high-dimensional space. The high-dimensional vectors are then fed into our proposed model CRISPR-HW (CRISPR model based on Hybrid Neural Network and Word embedding), we drew inspiration from ResNet and parallel connectivity network structures to address the potential loss of information due to data imbalance and deeper network layers. The encoding scheme and HNN model we offer outperform existing off-target prediction models not only on datasets with indels but also on multiple datasets with only mismatches.

## 2 Materials and methods

### 2.1 Datasets

To date, various off-target specificity assay studies have been performed, resulting in the publication of many publicly available off-target datasets that span different species and cell types and employ various in vitro and in vivo techniques. However, these datasets were not systematically organized, which limits our to comprehensively evaluate various aspects of model performance, so we performed a comprehensive review and compiled a total of ten different public datasets. These datasets were roughly divided into two types: the first type contains both mismatch and indel sequence pairs, while the second type contains only mismatch sequence pairs. For ease of identification, we have named each dataset and details of the datasets as shown in Table 1.

**Table 1:** Type 1 consists of samples that exhibit both mismatch and indel with 2 individual datasets and totaling approximately 800 000 samples, and Type 2 dataset solely focuses on mismatch patterns. It comprises eight distinct datasets, amounting to approximately one million samples.

Type	Technique	Dataset	Total Sample	Active Off-targets	Indels	gRNAs	Source
Type1	CIRCLE-Seq	CIRCLE	584 949	7 371	430	10	Tasi et al. [9]
	GUIDE-Seq	Listgarten_indel	213 943	60	13	6	Listgarten et al. [29]
Type2	PKD	Doench	4 853	2 273	-	65	Doench et al. [30]
	PDH	CRISPOR	10 129	354	-	19	Haeussler et al. [31]
	SITE-Seq	SITE	217 733	3 767	-	9	Cameron et al. [32]
	GUIDE-Seq	Tasi	294 534	52	-	9	Tasi et al. [11]
	GUIDE-Seq	Kleinstiver	95 829	54	-	5	Kleinstiver et al. [33]
	GUIDE-Seq	Listgarten	383 463	56	-	22	Listgarten et al. [29]
	Various assays	K562	18 434	118	-	12	Liu et al. [18]
	Various assays	Hek293t	57 245	515	-	18	Liu et al. [18]

Within the first type, there are two datasets: CIRCLE and Listgarten_indel. Tasi et al. [29] utilized CIRCLE-Seq technology to generate the CIRCLE dataset, which contains indels. On the other hand, Listgarten et al. [29] employed DUIDE-Seq technology to provide the Listgarten_indel dataset.

To comprehensively assess the model, we recognized the limited availability of off-target datasets specifically focused on indels. Consequently, we expand our evaluation by including multiple datasets that contains only mismatch. We gathered datasets comprising approximately one million sequence pairs from studies conducted by Haeussler, Cameron et al. Each dataset consists of pairs of sequences derived from multiple guide RNAs (gRNAs) [32], [31].

### 2.2 Analysis of datasets

In order to explore the impact of the aforementioned datasets on model performance, we analyzed the aforementioned datasets, a common feature of extreme imbalance between positive and negative samples was observed, as illustrated in Table 2. For instance, the Listgarten_indel dataset has a staggering positive to negative sample ratio of 4277:1, in scenarios where the majority class (negative samples) significantly outweighs the minority class (positive samples), a model becomes biased towards the majority class. As a result, the model achieves high accuracy due to its ability to accurately predict the majority class. However, this results in a low PR-AUC (Area Under the Curve) score, which is a metric primarily designed to assess the imbalance between positive and negative samples.

**Table 2:** Sample profiles for all datasets.

	Dataset	Total Sample	Negative Sample	Positive Sample	Sample Proportion
Type1	CIRCLE	584 949	577 578	7 371	78:1
	Listgarten_indel	213 933	213 883	50	4 277:1
Type2	Doench	4 853	2 580	2 273	1:1
	CRISPOR	10 129	10 077	52	193:1
	SITE	217 733	213 966	3 767	56:1
	Tasi	294 534	294 180	354	831:1
	Kleinstiver	95 829	95 775	54	1 773:1
	Listgarten	383 463	383 407	56	6 846:1
	K562	18 434	18 316	118	155:1
	Hek293t	57 245	56 730	515	110:1

In addition, we conducted an investigation into the impact of the position and type of base bulge on off-target effects. The dataset contains reads that reflected the current off-target activity. By analyzing the bulge position, the number of bulges, and the corresponding read of the off-target sequence pairs, we were able to establish a relationship between these factors.

**Fig. 2:**
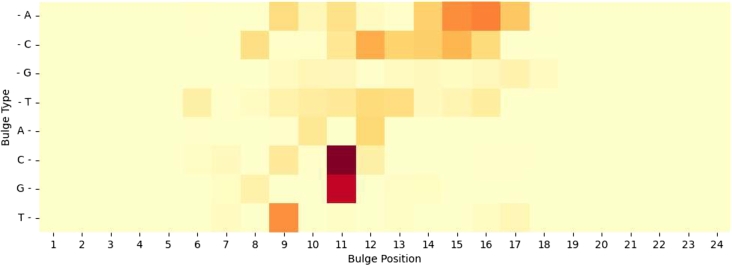
For each off-target sequence pair in the CIRCLE dataset, the number of reads of different base bulge types at different positions, with darker colors representing a greater effect on off-target activity.

Fig. 2 presents our findings regarding the effect of RNA bulges and DNA bulges on CIRCLE reads. It is evident from the figure that RNA bulges generally have a more significant impact on CIRCLE reads compared to DNA bulges. Moreover, the bulge positions are typically concentrated within the range of 8 bp to 20 bp away from the target site. Interestingly, bulges located near the PAM (Protospacer Adjacent Motif) end or the beginning of the sequence exhibit minimal influence on off-target reads.

### 2.3 Encoing gRNA-target sequences

**Fig. 3:**
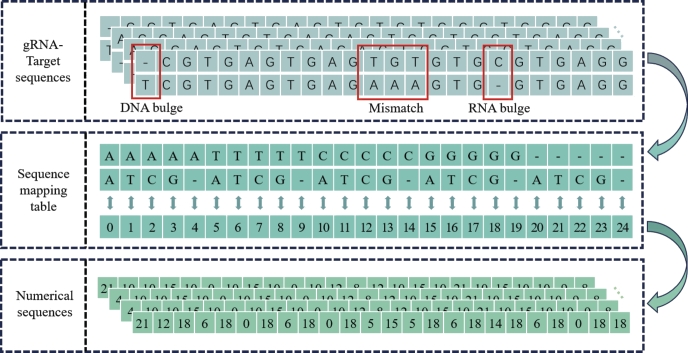
All gRNA-target sequences, encoded by sequence mapping table, are converted into unique tokens from 0-24.

In this study, our dataset consists of gRNA and DNA sequence pairs, with each sequence being 23 bases in length and composed of four standard bases (A, T, C, G). Each position within the gRNA-target sequence pair represents the formation of a base pair, which is considered a unique feature. When only mismatched sequence pairs are considered, four bases in the sequence pair can form 16 different base pairs (4*4). For sequence pairs containing indels, DNA and RNA bulges are marked with a ‘-’. As a result, 5 types of bases can form a total of 25 different base pairs (5*5). In order to encompass a wide range of sequences, we selected 25 base pair cases comprising the four canonical bases along with the ‘-’ symbol. These selected cases aimed to cover various types of base pairings, including DNA and RNA bulges as well as common mismatch occurrences. We utilized a mapping approach which named sequence mapping table to assign unique index values to 25 different base pairs of gRNA-target sequence (see Fig. 3).

Within our sequence mapping table, unique tokens are assigned to different types of base pairs variations encountered in the dataset. For instance, the RNA bulge ‘-A’ is represented by the token 21, while the DNA bulge ‘C-’ is denoted by the token 14. Similarly, for single nucleotide mismatches, we have assigned distinct tokens to different types of mismatches. For example, the mismatch ‘TA’ is mapped as 5, and the mismatch ‘GA’ is mapped as 15. By utilizing this sequence mapping table and the corresponding tokens, we can effectively capture various indels and mismatches within our datasets.

To the best of our knowledge, this is the first study to utilize the approach of word embedding to express sequence pairs containing indels. By doing so, we were able to convert the 25 types of base pairs into an 25 unique tokens. This tokens served as the input for the subsequent Embedding layer of model, allowing for efficient and accurate analysis.

### 2.4 CRISPR-HW model

**Fig. 4:**
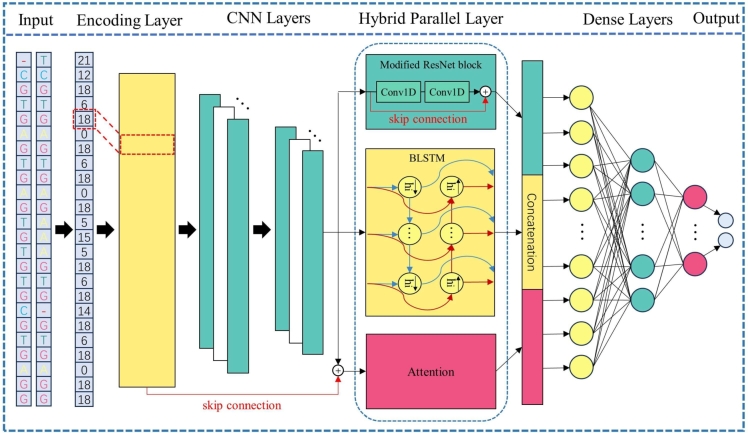
CRISPR-HW, an HNN model with a parallel network architecture. The model consists of four key components: the Sequence Encoding Layer, Feature Extraction Layer (CNN Layer), Hybrid Parallel Layer, and Dense Layer. One of the most important hybrid parallel layers contains optimized residual block, BLSTM and attention mechanisms.

We propose a HNN model, CRISPR-HW, based on parallel network architecture as shown in Fig. 4. This model is useful for tasks that require the model to capture the spatial and temporal dependencies in the input sequence. The proposed model comprises four components that work together to facilitate accurate off-target prediction: the Sequence Encoding Layer, Feature Extraction Layer, Hybrid Parallel Layer, and Dense Layer. Among them, the Hybrid Parallel Layer is the most important and contributes significantly to the accuracy of off-target prediction.

In the Sequence Encoding Layer, the data is preprocessed by mapping all base pairs (AA, AG, ..., TG, TT, –) formed by two bases at corresponding positions of gRNA-target to integers from 0 to 25, with each integer being a unique token. For example, ‘AA’ is mapped to 0, and ‘–’ is mapped to 25, resulting in a sequence of tokens of the same length as the input sequence. The token sequence is then converted into a dense vector of fixed size by an Embedding layer with a dimension of 90, which is inputted to the subsequent layer.

In the Feature Extraction Layer (CNN Layer), two simple convolution operations are performed on the dense vector to increase or decrease the dimension for local feature extraction. These convolutions use kernels of size 4 and 6, with corresponding dimensions of 70 and 40, respectively. A batch normalization is then applied after each convolution to improve training stability and prevent overfitting. The dimension of the convolution kernel is one-dimensional, and ReLU (Rectified Linear Unit) is chosen as the activation function. Batch normalization is performed after each convolution to speed up the convergence.

The Hybrid Parallel Layer comprises three components: a modified one-dimensional residual block, a Bidirectional Long Short-Term Memory (BLSTM) network, and an attention mechanism. We adopt distinct network models to achieve sequence perception in different dimensions, perform feature extraction, context modeling, and attentional focus. Ultimately, the outcomes of these three components are fused together. The three components are specified as follows:

1. The modified one-dimensional residual block not only learns the local information of the sequence and residual mapping to overcome the problem of losing some features of the original data after multiple layers and preventing gradient disappearance but also offers crucial advantages and unique contributions to HNN models. It excels in capturing local information within sequential data, preserving essential features, ensuring gradient stability during training, and maintaining consistent data size. These qualities make it a valuable asset in HNN models, particularly for tasks involving gene sequential data processing. The modified residual module contains two one-dimensional convolutions and batch normalization, to ensure consistent data size and maintain the integrity of the input, both convolutional filters in modified residual module have the same size, which is 5. ReLU is also chosen as the activation function before the final output.
2. Given that our data consists of sequences, we chose a BLSTM network, which is adept at handling the characteristics of sequential data and learning contextual features. It improves upon the traditional LSTM [25] architecture by comprising two LSTM layers: one processes the input sequence in a forward direction, while the other processes the output sequence in reverse. Each LSTM layer contains three essential network structures: the forget gate, input gate, and output gate. At each time step, the outputs of these two layers are concatenated to produce the final output sequence.
3. Based on the results of our data analysis (see Section 2.2) that varying base pairs at different positions can exert distinct effects on off-target outcomes, we have designed attention mechanisms [24] to assign higher weights to important positions and bases in the sequence, adaptively capture key features, and enhance feature interpretability. Additionally, we employ the concept of skip connections for residual networks to optimize the input data for the attention mechanism. This optimization is achieved by fusing the outputs of two layers: the dense vector generated by the Embedding layer and the dimensional processing layer. By effectively fusing the outputs from different parts of the model, the attention mechanism can extract more meaningful and representative features, resulting in improved performance in tasks such as off-target prediction in CRISPR gene editing.

The outputs of the three parallelized models are flattened and used as inputs to the Dense layer. This component consists of three dense layers comprising 300, 100, and 2 neurons, respectively. In order to increase the nonlinear relationship between dense layers, we apply ReLU as the activation function of the first two dense layers. In the first two dense layers, dropout regularization is implemented to enhance the model's generalization ability and prevent overfitting, and the parameter is set to 0.2 in this work. This regularization technique effectively reduces the model's reliance on specific features, promoting the learning of more robust and generalized representations. In the last dense layer, comprising two neurons, utilizes the softmax activation function to assign probabilities to the off-target outcomes.

### 2.5 Experimental settings and model parameters

The experimental environment for our published models was implemented using Python 3.7.16 and utilized the following libraries: Tensorflow (version 2.2.0) and Keras (version 2.3.0). To ensure the consistency and reliability of all experimental results, all computations were performed on a dedicated server (Dell Precision 3660). The server's GPU was equipped with an NVIDIA GeForce GTX 4090, which offered 24 GB of memory.

To ensure a robust evaluation and mitigate the impact of data variability, we employ a StratifiedKFold class initializes a StratifiedKFold object with 5 splits (folds). Firstly, the dataset used in our study is shuffled to randomize the sample order and then divided into five equal parts. During the 5-fold cross-validation process, four parts serve as the training set, while the remaining part acts as the validation set. This process is repeated five times, with each part being used as the validation set once. Finally, we obtain five sets of model performance metrics. By calculating the average performance metrics across these five results, we obtain a more comprehensive and representative assessment of the model's effectiveness in predicting off-target effects.

For the model, the loss function is set to Categorical Crossentropy, which is commonly applied for multi-class classification tasks. The optimizer chosen is Adam, known for its effectiveness in optimizing the loss function. Additionally, a ReduceLROnPlateau callback is created. This callback monitors the validation loss (monitor=‘val_loss’) and reduces the learning rate (reduce_lr) when the validation loss plateaus. Specifically, if there is no improvement in the validation loss for 3 consecutive epochs (patience=3), the learning rate is decreased by a factor of 0.2 (factor=0.2). The minimum learning rate is set to 1e-5 (min_lr=1e-5). It is important to note that the initial learning rate is set to 0.003 to initiate the model training process. In addition, in the comparison of different prediction models (Section 3.2), uniform epoch settings can result in biased outcomes since models inherently exhibit varied convergence speeds. Therefore, we individually tailored the epoch and batch size for each model based on their unique convergence properties.

### 2.6 Model selection for ablation experiments

To evaluate the effectiveness of the parallel network architecture, we conducted a series of ablation experiments. In these experiments, we selectively blocked different parts of our HNN model to create five ablation models (see Table 3). We compared the performance of these models using two types of datasets: the Listgarten_indel dataset and the SITE dataset. By comparing their performance against the original model, we can determine the usefulness and contribution of each module in enhancing the model's effectiveness and robustness.

**Table 3:** Details of the four ablation models in the ablation experiment.

model	Architecture
CRISPR-HW	Original model
CRISPR-HW-NO-BLSTM	Without BLSTM
CRISPR-HW-NO-ResNet	Without ResNet
CRISPR-HW-NO-Attention	Without Attention mechanism
CRISPR-HW-NO-Dense	Without Dense Layers
CRISPR-HW-Serial	Without parallel structure

The CRISPR-HW-NO-ResNet model is created by removing the modified residual network module from the parallel layer. The CRISPR-HW-NO-BLSTM model involves blocking the BLSTM component of the original model while keeping the remaining model parameters unchanged. This enables us to observe whether the HNN model's ability to learn sequential contextual information significantly enhances the overall performance of the model. The CRISPR-HW-NO-Attention model is derived by removing the attention mechanism from the original model. By evaluating the performance of this model, we can assess the crucial role of the attention mechanism in assigning different weights to different positions in the sequence. Lastly, the CRISPR-HW-NO-Dense model eliminates the Dense layer from the original model. This allows us to assess the impact of the Dense layer on the overall effectiveness of the model. Finally, the CRISPR-HW-Serial is a transformation of the model's parallel network structure into a serial network structure, where the advantages of the parallel network structure can be demonstrated.

### 2.7 Performance evaluation

Given the unbalanced nature of our datasets (refer to Section 2.2), relying on accuracy as a performance metric can potentially lead to misleading and thus prevent a comprehensive evaluation of the model [34], [35]. Consequently, we have opted for a suite of metrics, including PR-AUC, ROC-AUC and Recall.

In particular, we emphasize the importance of Precision-Recall Area Under the Curve (PR-AUC) metrics due to its X-axis denotes the recall, otherwise known as the true positive rate or sensitivity, and the Y-axis represents precision. Recall serves to calculate the proportion of actual positive samples correctly identified as such, and precision evaluates the percentage of predicted positive samples that are genuinely positive. PR curves were constructed and PR-AUC values were computed by establishing various thresholds. Relative to other metrics, in the case of positive and negative sample imbalance, we can visualize and quantify more intuitively the performance of the model at different levels of precision and recall. The respective mathematical calculations for this metrics are elucidated as follows:

(1) $$Recall=\frac{TP}{TP+TN}$$

(2) $$Precision=\frac{TP}{TP+FP}$$

## 3 Results

In this section, we initially adapt the Sequence Encoding Layer of the CRISPR-HW model, transitioning it to the one-hot encoding scheme employed by CRISPR-Net. We subsequently validate the efficacy of CRISPR-HW's encoding model across diverse datasets. Secondly, we identify models demonstrating superior predictive performance on various datasets for comparison with CRISPR-HW. Then, we utilize ablation experiments to authenticate the contribution of individual components of the CRISPR-HW model towards enhancing predictive performance. Lastly, we examine the model's generalizability through the prediction of disparate datasets using the trained model.

### 3.1 Evaluation of encoding schemes

To showcase the effectiveness of our encoding scheme when applied to off-target datasets, we modified the Sequence Encoding Layer to accommodate two different one-hot coding schemes, C2 and C3. These schemes were proposed by Zhang et al. [36] and Lin et al. [16], respectively. C1 is our original coding scheme. Given that the vector sizes produced by the word embedding coding scheme and the one-hot coding scheme differ, we made corresponding modifications to the CNN Layer to accommodate these two coding schemes, while the remainder of the model was left unchanged.

As depicted in Table 4, our coding scheme C1 yields the highest PR_AUC (0.5858) and ROC_AUC (0.9874) on the indel-containing CIRCLE dataset, indicating a 7% improvement over the PR_AUC of C2 (0.5467) and a 3% enhancement compared to C3 (0.5671). Regarding the three datasets containing only off-targets, the PR_AUC (0.8016) on the SITE dataset surpasses the suboptimal C2 by 5.1%, and the PR_AUC on the K562 dataset (0.8341) exceeds the next best, C3, by 3.3%. Although the PR_AUC on the Hek293t dataset (0.8103) does not achieve the highest rating, it trails the top result, C3, by a narrow margin of 0.4%. Due to data imbalance, the ROC_AUC for all coding methodologies are comparatively similar, each exceeding 0.98. In summary, our novel coding scheme contributes to efficacious feature extraction and prediction for datasets containing indels, as well as datasets comprising only mismatches.

**Table 4:** C1 is the coding scheme used in our original model, C2 is the latest coding scheme proposed by Zhang et al. [36], and C3 is the coding scheme proposed by Lin et al. [16]. All results are averages from a 5-fold cross-validation, and effects that stand out are bolded.

Metrics	Encoding	CIRCLE	SITE	Hek293t	K562
PR_AUC	C1	**0.5858**	**0.8016**	0.8064	**0.8341**
	C2	0.5467	0.7626	0.7638	0.7656
	C3	0.5671	0.7479	**0.8103**	0.8072
ROC_AUC	C1	**0.9874**	**0.9889**	0.9899	0.9933
	C2	0.9689	0.9858	**0.9972**	0.9926
	C3	0.9717	0.9838	0.9924	**0.9970**

### 3.2 Comparison of different models

In this section, we conduct a comparative analysis between the CRISPR-HW model and the prevailing state-of-the-art models for off-target predictions, employing two distinct types of datasets.

**Fig. 5:**
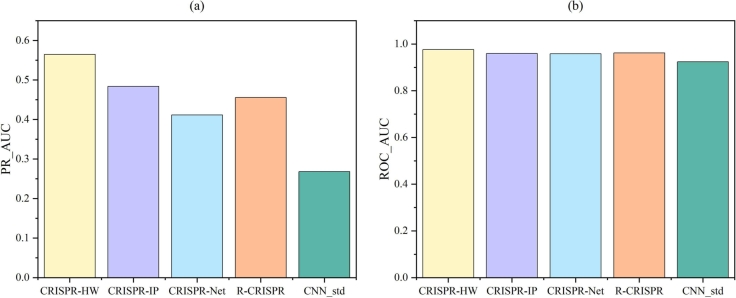
Comparison of performance of CRISPR-HW with four existing off-target prediction models on the CIRCLE dataset in terms of PR_AUC (a) and ROC_AUC (b). The values represent the average of a 5-fold cross-validation.

Firstly, we chose three prediction models that perform excellently in the CIRCLE dataset, namely CRISPR-IP [36], CRISPR-Net [16], R-CRISPR [37], and another modified CNN_std from the CRISPR-IP paper [36]. The experimental results of the comparison between CRISPR-HW and these four models are shown in Fig. 5. CRISPR-HW demonstrates superior performance over the other four models, achieving a mean PR_AUC of 0.5648 and ROC_AUC of 0.9764. This showcases an enhancement of 16.7% in PR_AUC and a 1.7% uptick in ROC_AUC in comparison to the current front-runner, CRISPR-IP.

**Fig. 6:**
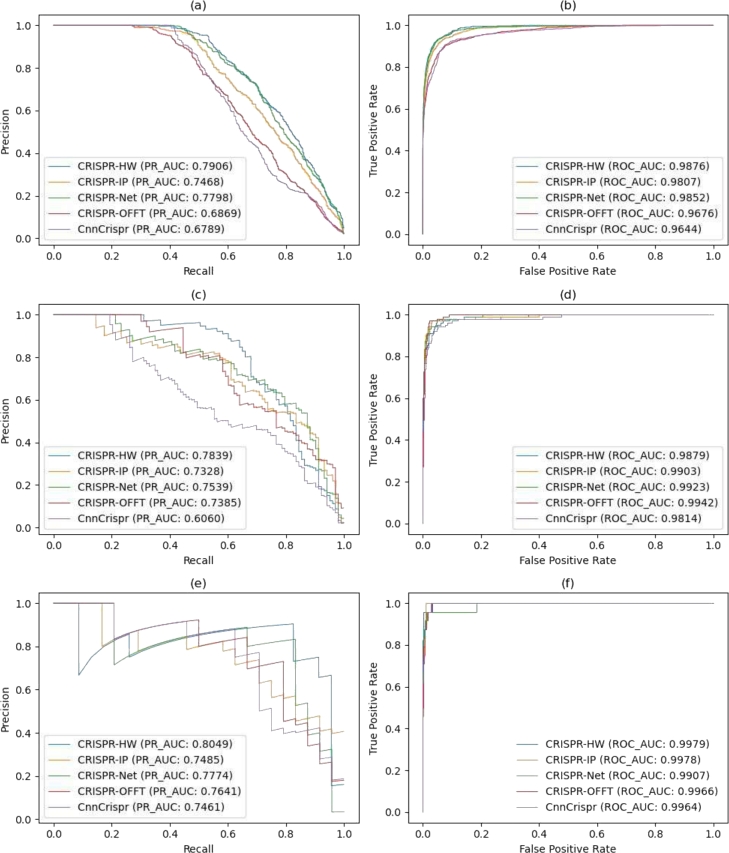
Comparison of the performance of CRISPR-HW with 4 existing off-target prediction methods in the SITE dataset, PR_AUC in part (a) and ROC_ AUC in part (b). Part (c) with PR_AUC and part (d) with ROC_AUC present a comparative performance evaluation of CRISPR-HW against four current off-target prediction models using the Hek293t dataset. Part (e) presents PR_AUC and part (f) displays ROC_AUC, both of which illustrate a comparison of the model's performance on the K562 dataset.

Furthermore, to demonstrate the predictive capability of our model specifically on datasets containing only mismatches, we selected the SITE, Hek293t, and K562 datasets. We drew comparisons with four other preeminent existing models: CRISPR-IP, CRISPR-NET, CRISPR-OFFT [20], and CnnCrispr [18]. The results of this comparative evaluation are depicted in Fig. 6. Fig. 6(a) and 6(b) present the comparative results of our model on the SITE dataset. As shown, in both PR_AUC and ROC_AUC evaluations, CRISPR-HW's performance surpasses that of the other four models, achieving values of 0.7906 and 0.9876, respectively. The PR_AUC exceeds that of CRISPR-Net by 1.3% and surpasses the lowest performer, CnnCrispr, by 16.4%. Regarding ROC_AUC, our model's performance is comparable to that of CRISPR-IP and CRISPR-Net. Fig. 6(c) and 6(d) display the PR and ROC curves for the Hek293t dataset, respectively. Our model delivers the highest PR_AUC results, surpassing the suboptimal CRISPR-Net by 3.9% and outperforming the least effective model, CnnCrispr, by 29.3%. Although our model's ROC_AUC does not achieve the highest score for this dataset, it falls short of the top performer, CRISPR-IP, by a mere 0.0063. Regarding the K562 dataset, as illustrated in Fig. 6(e) and 6(f) for PR_AUC and ROC_AUC respectively, CRISPR-HW exhibits a slight advantage, noting approximately a 3.5% and 5.4% improvement over CRISPR-OFFT and CRISPR-Net. Given that this dataset is characterized by a smaller sample size, the ROC_AUC for all models exceeds 0.99. In conclusion, our model demonstrates competitiveness in the prediction of off-target activity for datasets consisting solely of mismatches.

### 3.3 Ablation experiments

In this section, we undertake ablation experiments to assess the influence of each component of the model on the predictive outcome. The construction of the ablation model and the selection of the dataset stem from Section 2.6.

Firstly, as shown in Table 5, in the Listgarten_indel dataset, which contains indel, we note that the omissions of the attention mechanism (CRISPR-HW-NO-Attention) and the Dense layers (CRISPR-HW-NO-Dense) exert the most significant impact on our original model, with PR_AUC diminishing by 13.2% and 21.1%, respectively. While the ROC_AUC value for CRISPR-HW (0.9781) isn't the highest, it only trails by 0.8% compared to CRISPR-HW-NO-Dense (0.9862), which has the best performance. The best recall value was observed for CRISPR-HW, outperforming the CRISPR-HW-NO-BLSTM, by 28.5%, and exceeding other ablation models by 12.4%.

**Table 5:** The results of the ablation experiments on the two datasets used a total of six ablation models and three evaluation metrics.

Dataset	Model	PR_AUC	ROC_AUC	Recall
Listgarten_indel	CRISPR-HW	**0.2827**	0.9781	**0.1800**
	CRISPR-HW-NO-BLSTM	0.2636	0.9672	0.1400
	CRISPR-HW-NO-ResNet	0.2786	0.9797	0.1600
	CRISPR-HW-NO-Attention	0.2497	0.979	0.1600
	CRISPR-HW-NO-Dense	0.2334	**0.9862**	0.1600
	CRISPR-HW-Serial	0.2682	0.9745	0.0800
Hek293t_K562	CRISPR-HW	**0.7412**	**0.9905**	**0.6051**
	CRISPR-HW-NO-BLSTM	0.7252	0.9894	0.5861
	CRISPR-HW-NO-ResNet	0.7212	0.9875	0.5767
	CRISPR-HW-NO-Attention	0.7289	0.9885	0.6002
	CRISPR-HW-NO-Dense	0.7334	0.9887	0.5640
	CRISPR-HW-Serial	0.7004	0.9857	0.5688

By examinating mismatch-only datasets Hek293t and K562, the removal of the modified residual block, the BLSTM, and the Attention mechanism from the parallel network structure resulted in declines of 2.2%, 2.7%, and 1.6% in PR_AUC, respectively. The removal of Dense layers had the least impact on the model's performance, causing only a 1% drop in PR_AUC, but interestingly, it could adversely influence the model's Recall. The transformation from a parallel network structure to a serial one yields the most significant impact on the overall model, manifesting the lowest PR_AUC (0.7004) and ROC_AUC (0.9857) values in the overall ablation model prediction performance, with reductions of 5% and 0.4%, respectively.

The results elucidate the capacity of various components within the parallel network structure to discern distinct features from the sequence data, collectively fostering an augmentation in the model's comprehensive performance.

### 3.4 Generalization capability of the model

**Fig. 7:**
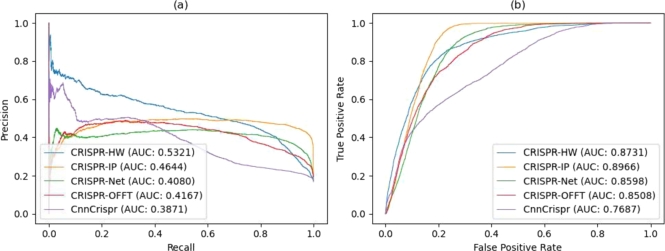
Comparison of the generalization performance of CRISPR-HW with four other off-target prediction models on the Doench and CRISPOR datasets. All models were trained on the Tasi, Kleinstiver, and Listgarten datasets.

To ensure our model's adaptability to various off-target data, we conducted experiments to verify the generalization ability of CRISPR-HW. The model was trained on the Tasi, Kleinstiver, and Listgarten datasets, all of which were sourced using the GUIDE-Seq technique. Due to the sparse sampling of the Doench and CRISPOR datasets, we consolidated these two for model testing. As depicted in the ROC and PR curves in Fig. 7(a) and Fig. 7(b), our model achieved the highest PR_AUC (0.5321), surpassing the second-place CRISPR-IP by 14.5% and outperforming CnnCrispr by 37.4%. Furthermore, our model exhibited commendable performance in ROC_AUC, attaining a value of 0.8731, it has improved by 13.5% compared to the ROC_AUC (0.7687) of CnnCrispr. Although the model did not achieve the highest rank in ROC_AUC, the PR_AUC offers a more comprehensive evaluation of its performance, particularly considering the imbalanced nature of the off-target data, a higher PR_AUC value means that the model is more adaptive to unbalanced datasets. As such, the aforementioned results illustrate that CRISPR-HW outperforms other models in off-target prediction, thereby showcasing its robust generalization capability.

## 4 Discussion and conclusion

The primary objective of this study was to develop a predictive model for off-target effects in CRISPR-Cas9 gene editing, aiming to elucidate the underlying mechanisms. To address this challenge, we introduced an innovative approach that utilized a word-embedded coding scheme for the representation of off-target data, encompassing both mismatches and indels. This approach effectively streamlines the intricacies associated with sequence encoding while minimizing information loss throughout the coding process. Furthermore, our model employs a hybrid parallelization strategy to accommodate the diverse features inherent in these sequences. Within this framework, an optimized residual block is incorporated into modules of parallel layers. This strategic integration aims to closely align the model with our input sequences, thus mitigating the potential loss of sequence information as it traverses through the model's layers. To capture essential contextual features, we employ Bidirectional Long Short-Term Memory (BLSTM) units. Additionally, we incorporate an attention mechanism, which aids the model in concentrating on critical base changes during the analysis process. This multifaceted approach contributes to a comprehensive and precise assessment of off-target effects in CRISPR-Cas9 gene editing, enhancing our understanding of this crucial mechanism.

Our experiments compared our word embedding scheme with the one-hot coding schemes used in previous models, demonstrating that our coding method delivers competitive performance across two types of datasets. Compared to existing deep learning models, our model excels in both PR_AUC and ROC_AUC metrics, outperforming four pre-existing off-target prediction models across four independent datasets. This illustrates the effectiveness of our model. Through ablation experiments, we highlighted the positive influence of different network models within the HNN model on the overall results. Finally, we conducted a generalizability evaluation to show that CRISPR-HW can accurately predict untrained sequences, our model, trained solely on the dataset obtained through the GUIDE-Seq technique, performs competitively when predicting off-target effects in other sequence pairs. In summary, this study has not only applied word embedding to off-target datasets containing indels but also proposed a more competitive off-target prediction model. This provides new insights into the study of off-target mechanisms in the CRISPR-Cas9 gene editing system, offering a novel perspective for the field of deep learning within bioinformatics. However, it's important to note that the generalization ability of existing models continues to be hampered by sample imbalance [38], [39], and the precise mechanism of off-target effects in gene editing remains to be deciphered [40]. These challenges will be the focal points of our future research.

## Funding

The paper is supported by Key R&D Projects in Zhejiang Province (2022C02044, 2022C02009).

## CRediT authorship contribution statement

**Yanpeng Yang:** Conceptualization, Investigation, Methodology, Writing – original draft. **Jian Li:** Data curation, Formal analysis, Writing – original draft. **Quan Zou:** Resources, Supervision. **Yaoping Ruan:** Visualization, Writing – review & editing. **Hailin Feng:** Conceptualization, Funding acquisition, Resources, Supervision, Writing – review & editing.

## Declaration of Competing Interest

The authors declare that they have no known competing financial interests or personal relationships that could have appeared to influence the work reported in this paper.

## Data Availability

Supporting datasets and source codes for this study are readily accessible at the following location: https://github.com/Yang-k955/CRISPR-HW.
